# Analysis of the complete mitochondrial genome sequence of Japanese butterflyfish, *Chaetodon nippon* (Chaetodontiformes, Chaetodontidae)

**DOI:** 10.1080/23802359.2023.2185080

**Published:** 2023-03-13

**Authors:** Maheshkumar Prakash Patil, Jong-Oh Kim, Yu-Jin Lee, Yong Bae Seo, Jin-Koo Kim, Gun-Do Kim

**Affiliations:** a Industry-University Cooperation Foundation, Pukyong National University, Busan, Republic of Korea; b Department of Microbiology, Pukyong National University, Busan, Republic of Korea; c School of Marine and Fisheries Life Science, Pukyong National University, Busan, Republic of Korea; d Department of Marine Biology, Pukyong National University, Busan, Republic of Korea; e Research Institute for Basic Science, Pukyong National University, Busan, Republic of Korea

**Keywords:** *Chaetodon nippon*, Japanese butterflyfish, Chaetodontidae, mitogenome, phylogenetic analysis

## Abstract

Japanese butterflyfish (*Chaetodon nippon*) belong to the family Chaetodontidae and order Chaetodontiformes. It has circular mitochondrial genome of 16,507 bp in length with 55.4% of A + T content and has 37 genes, including 22 tRNA, 2 rRNA, and 13 protein-coding genes, in addition to a control region. The results of phylogenetic analysis indicated that the *C. nippon, C. wiebeli*, *C. auripes*, *C. auriga*, *C. octofasciatus*, *C. speculum*, and *C. modestus* are closely related to each other. The findings of this study will provide useful genetic information for further phylogenetic and taxonomic classifications of Chaetodontidae.

## Introduction

Japanese butterflyfish (*Chaetodon nippon,* Steindachner and Doederlein, 1883) belongs to the family Chaetodontidae and order Chaetodontiformes and can be found on shallow rocky reefs, off the shores of the Philippines, South Korea, Taiwan, and Japan. Fishes belonging to the family Chaetodontidae serve as an indicator fish group for the coral reef environment. The fish species in the genus *Chaetodon* are widely distributed; however, they are closely related based on morphology and selected mitochondrial genes (Hsu et al. 2007; Ferdyan et al. 2021). Mitogenomes have been widely used as molecular markers for evolutionary phylogeny and population genetics studies in a broad range of species. While no previous research on the complete mitogenome of *C. nippon* has been reported, we report the complete mitochondrial genome sequencing of *C. nippon* and its phylogenetic relationship with other *Chaetodon* species. The mitogenome sequence of *C. nippon* reported in this study will provide useful genetic data for species identification; the understanding of taxonomic classification, phylogenetic relationship, and the evolutionary history of the Chaetodontidae.

## Materials

**Figure 1.**
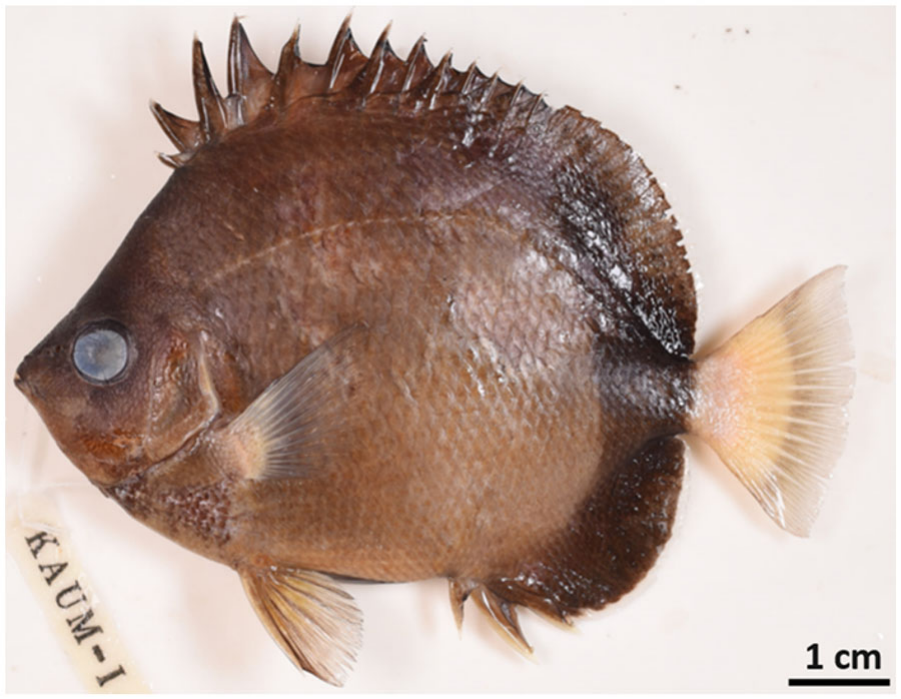
Japanese butterflyfish (*Chaetodon nippon*) (Photo by Yu-Jin Lee). The body of this species is creamy-brown with a dark brown rim and a spiny dorsal fin.

The *C. nippon* specimen was donated by Professor Hiroyuki Motomura, Kagoshima University Museum, Japan. It was collected from the coast of Uchinoura Bay in Japan (31°29′03.59″N 131°07′89.57″E) (Figure 1). The specimen was deposited under the voucher number KAUM-131676 at the Marine Fish Resources Bank of Korea (MFRBK), Pukyong National University (PKNU), Busan, South Korea (Jin-Koo Kim, taengko@pknu.ac.kr).

## Methods

Total genomic DNA was extracted from the muscle using the DNeasy Blood and Tissue Kit (Qiagen, Germany) according to the manufacturer’s instructions. The TrueSeq Nano DNA Kit was used to construct the DNA library, which was then sequenced with 150 bp paired-end reads on the Illumina platform (Illumina, HiSeq 2500, San Diego, CA, USA). The obtained reads were cleaned using cutadapt 1.9 (Martin 2011), and the low-quality reads (*Q* < 20) were removed. As shown in Figure S1, using SPAdes v3.13.0, the cleaned sequences were used for *de novo* assembly (Bankevich et al. 2012). Protein-coding, rRNA and tRNA genes were annotated using MitoFish (http://mitofish.aori.u-tokyo.ac.jp/) (Iwasaki et al. 2013)

Complete mitochondrial genome sequences of *C. nippon*, 11 additional species from the family Chaetodontidae found in GenBank, and three species as outgroup members (*Leiognathus brevirostris* and *L. ruconius* from Leiognathidae and *Salvelinus malma* from Salmonidae) were used to construct a phylogenetic tree (Table 1). Prior to analysis, several mitochondrial genome sequences were aligned using ClustalW. Using MEGA11 v11.0.8 (Tamura et al. 2021) and MrBayes v3.2.6 (Huelsenbeck and Ronquist 2001), the phylogenetic tree was constructed using maximum likelihood (ML) with 1000 bootstrap.

**Table 1.** A list of the GenBank accession numbers of the complete mitochondrial genome sequences used in this study.

Species	GenBank Accession No.	References
*Chaetodon wiebeli*	MN170515	Yukai et al. (2019)
*Chaetodon auripes*	AP006004	Yamanoue et al. (2007)
*Chaetodon auriga*	ON843633	Patil et al. (2022a)
*Chaetodon speculum*	MW363870	Yang et al. (2021)
*Chaetodon octofasciatus*	MH094803	Zhu et al. (2018)
*Chaetodon nippon*	ON843632	In this study
*Chaetodon modestus*	ON843631	Patil et al. (2022b)
*Chelmon rostratus*	KM978959	Wang et al. (2016)
*Forcipiger flavissimus*	MZ329988	NA
*Heniochus chrysostomus*	MW136414	Yu et al. (2021)
*Heniochus diphreutes*	AP006005	Yamanoue et al. (2007)
*Heniochus acuminatus*	MW039154	Jiang et al. (2022)
*Salvelinus malma*	MF680544	Yang et al. (2017)
*Leiognathus brevirostris*	MN912698	NA
*Leiognathus ruconius*	MN251862	Sui et al. (2019)

## Results

**Figure 2.**
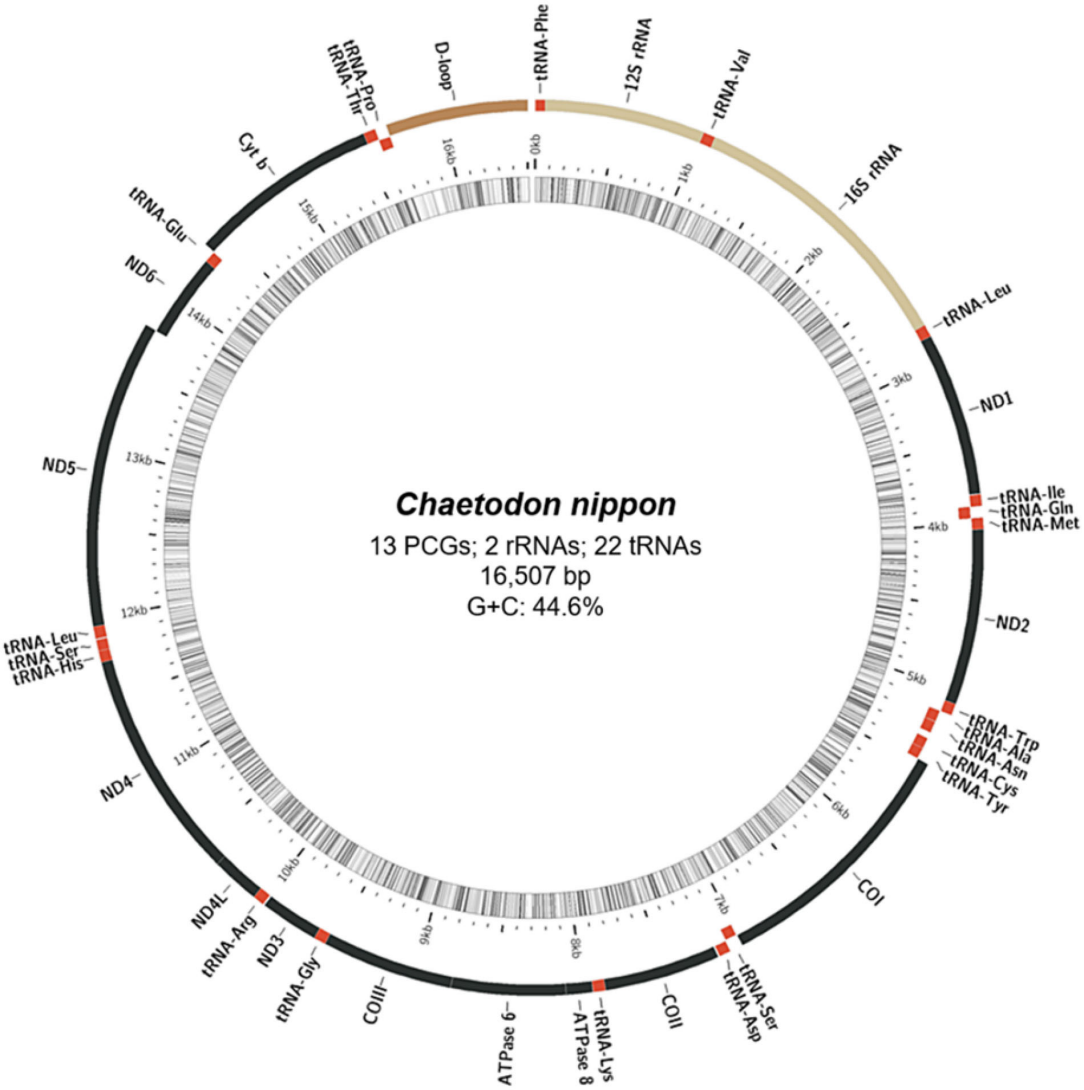
Gene map of the mitochondrial genome of Japanese butterflyfish (*Chaetodon nippon*). Genes outside the circle are transcribed clockwise, whereas those inside are transcribed anticlockwise. Using MitoFish (http://mitofish.aori.u-tokyo.ac.jp/), a circular map was created by applying annotation results. Regions of protein-coding genes (PCGs) marked in black, tRNA in red, rRNA in yellow, and control region (D-loop) in brown.

**Figure 3.**
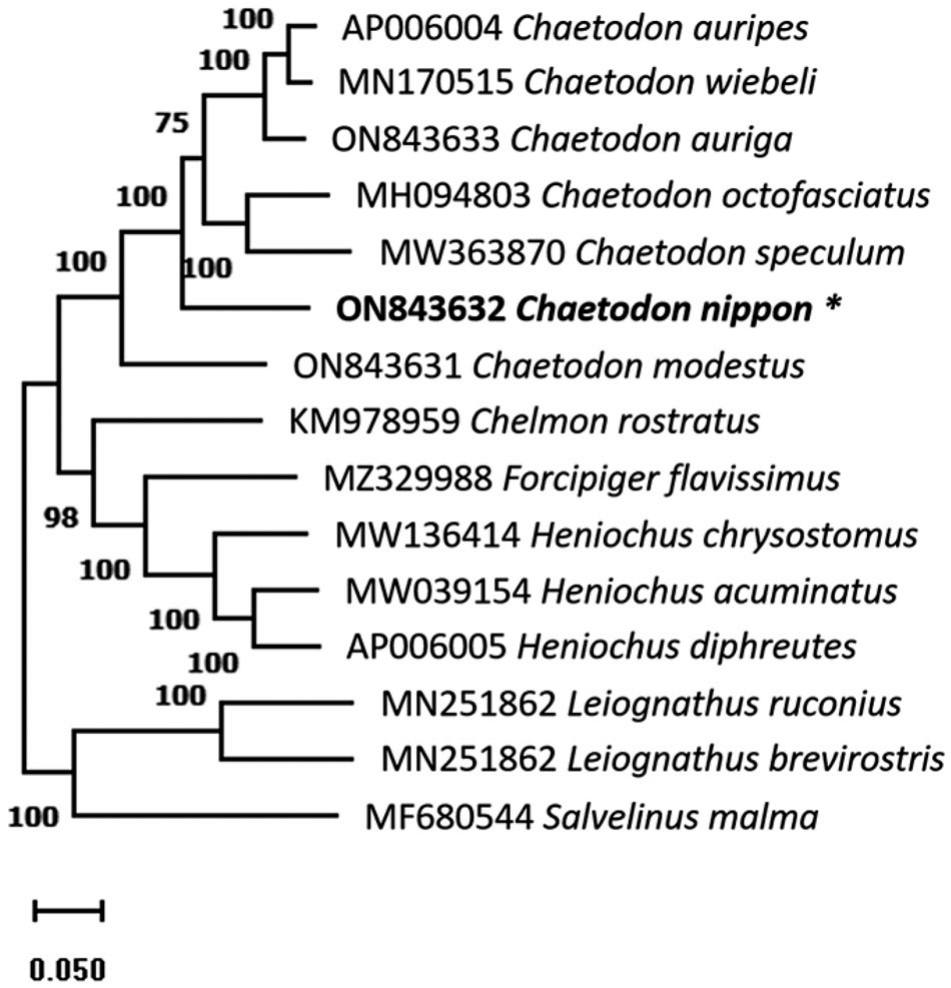
The phylogenetic tree was constructed using whole mitochondrial genome sequences of 15 species based on the maximum likelihood with 1000 bootstrap replicates using MEGA11. The bootstrap support values are shown by the numbers on the branches, and the species in this study is indicated by an asterisk next to its name.

The annotated complete mitochondrial genome sequence of *C. nippon* was submitted to NCBI (GenBank: ON843632). It was 16,507 bp in length, containing 37 genes, including 13 protein-coding, 2 rRNA and 22 tRNA genes, and a control region (D-loop, Figure 2). The overall base composition was 27.5%, 27.9%, 16.8%, and 27.8% for A, T, G, and C, respectively, with a slight A + T bias (55.4%). The 12S and 16S rRNA genes of *C. nippon* were positioned between the tRNA-Phe and tRNA-Leu genes, separated by the tRNA-Val gene. Additionally, several protein-coding genes, including *ATP6*, *COX2*, *COX3*, *CYTB*, *ND2*, and *ND4*, had incomplete stop codons. The ML phylogenetic trees constructed using MEGA11 (Figure 3) and MrBayes (Figure S2) were based on the complete mitochondrial genome sequences, showed that *C. nippon* is monophyletic with *C. wiebeli, C. auripes, C. auriga, C. octofasciatus, C. speculum*, and *C. modestus*.

## Discussion and conclusion

The characteristic features of the *C. nippon* mitochondrial genome, such as A + T content, positions of 12S and 16S rRNA, and incomplete stop codons of protein-coding genes, are in good accordance with those of other Chaetodontidae species (Zhu et al. 2018; Yukai et al. 2019; Patil et al. 2022a, 2022b). The lengths of the mitochondrial genome and control region vary among *Chaetodon* species (Table S1). The difference in length has become a benchmark for research on interspecific genetic diversity, which may result in phylogenetic reconstructions of numerous closely related species. The phylogenetic tree shows that *Chaetodon* species are monophyletic and closely related, which is consistent with previous studies (Patil et al. 2022a, 2022b). The findings of this study may help future population genetics and evolution studies.

.

## Ethical approval

The sample used for this study was a dead body of fish and as per the animal experimental ethics in the Republic of Korea (Standard operating guideline; IACUC – Institutional Animal Care and Use Committee, Book no. 11-1543061-000457-01, effective from Dec. 2020) does not need any approval from an Ethics Committee.

## Supplementary Material

Supplemental Material

## Data Availability

The genome sequence data that support the findings of this study are openly available in GenBank of NCBI at (https://www.ncbi.nlm.nih.gov/nuccore/ON843632) under accession no. ON843632. The associated BioProject, BioSample, and SRA numbers are PRJNA854332, SAMN29431623, and SRR19905897, respectively

## References

[CIT0001] Bankevich A, Nurk S, Antipov D, Gurevich AA, Dvorkin M, Kulikov AS, Lesin VM, Nikolenko SI, Pham S, Prjibelski AD, et al. 2012. SPAdes: a new genome assembly algorithm and its applications to single-cell sequencing. J Comput Biol. 19(5):455–477. 22506599 10.1089/cmb.2012.0021 PMC3342519

[CIT0002] Ferdyan R, Razak A, Ahda Y. 2021. Comparison of the Chaetodontidae mitochondrial genome. Biota: biologi Dan Pendidikan Biologi. 14(1):46–55.

[CIT0003] Hsu KC, Chen JP, Shao KT. 2007. Molecular phylogeny of Chaetodon (Teleostei: Chaetodontidae) in the Indo-West Pacific: evolution in geminate species pairs and species groups. Raffles Bull Zool. 14:77–86.

[CIT0004] Huelsenbeck JP, Ronquist F. 2001. MRBAYES: Bayesian inference of phylogenetic trees. Bioinformatics. 17(8):754–755. 11524383 10.1093/bioinformatics/17.8.754

[CIT0005] Iwasaki W, Fukunaga T, Isagozawa R, Yamada K, Maeda Y, Satoh TP, Sado T, Mabuchi K, Takeshima H, Miya M, et al. 2013. MitoFish and MitoAnnotator: a mitochondrial genome database of fish with an accurate and automatic annotation pipeline. Mol Biol Evol. 30(11):2531–2540. 23955518 10.1093/molbev/mst141 PMC3808866

[CIT0006] Jiang F, Yang N, Huang H. 2022. Characterization and phylogenetic analysis of the mitochondrial genome sequence of *Heniochus acuminatus*. Mitochondrial DNA Part B Resour. 7(9):1694–1695. 10.1080/23802359.2022.2049016 PMC9518272 36188664

[CIT0007] Martin M. 2011. Cutadapt removes adapter sequences from high-throughput sequencing reads. EMBnet J. 17(1):10–12.

[CIT0008] Patil MP, Kim J-O, Lee Y-J, Seo YB, Kim J-K, Kim G-D. 2022a. The complete mitochondrial genome of threadfin butterflyfish, *Chaetodon auriga* (Chaetodontiformes: chaetodontidae) and phylogenetic analysis. Mitochondrial DNA Part B Resour. 7(11):1922–1924. 10.1080/23802359.2022.2136982 PMC9639548 36353060

[CIT0009] Patil MP, Kim J-O, Lee Y-J, Seo YB, Kim J-K, Kim G-D. 2022b. Complete mitochondrial genome of brown-banded butterflyfish *Chaetodon modestus* (Chaetodontiformes, Chaetodontidae) and phylogenetic analysis. Mitochondrial DNA Part B Resour. 7(11):2012–2014. 10.1080/23802359.2022.2148490 PMC9704067 36451967

[CIT0010] Sui Y, Qin B, Song X, Sheng W, Zhang B. 2019. Complete mitochondrial genome of the deep pugnose ponyfish *Secutor ruconius* (Perciformes: Leiognathidae) in the East China Sea. Mitochondrial DNA Part B Resour. 4(2):3563–3564. 10.1080/23802359.2019.1670108 PMC7707339 33366086

[CIT0011] Tamura K, Stecher G, Kumar S. 2021. MEGA11: molecular evolutionary genetics analysis version 11. Mol Bio Evol. 38(7):3022–3027. 33892491 10.1093/molbev/msab120 PMC8233496

[CIT0012] Wang LJ, You F, Wu ZH. 2016. Complete mitochondrial genome of copperband butterflyfish *Chelmon rostratus* (Teleostei, Perciformes, Chaetodontidae). Mitochondrial DNA Part A DNA Mapp Seq Anal. 27(3):2141–2142. 10.3109/19401736.2014.982596 25427816

[CIT0013] Yamanoue Y, Miya M, Matsuura K, Yagishita N, Mabuchi K, Sakai H, Katoh M, Nishida M. 2007. Phylogenetic position of tetraodontiform fishes within the higher teleosts: Bayesian inferences based on 44 whole mitochondrial genome sequences. Mol Phylogenet Evol. 45(1):89–101. 17490896 10.1016/j.ympev.2007.03.008

[CIT0014] Yang Y, Li T, Lin H, Huang X, Yu W, Huang Z. 2021. The complete mitochondrial genome of *Chaetodon speculum* (Chaetodontiformes, Chaetodontidae). Mitochondrial DNA Part B Resour. 6(4):1290–1291. 10.1080/23802359.2021.1906170 PMC8018470 33855180

[CIT0015] Yang LG, Meng FX, Wang RX, Shi G. 2017. Complete mitochondrial genome of the *Salvelinus malma* sp. (Salmoniformes, Salmonidae) with phylogenetic consideration. Mitochondrial DNA Part B Resour. 2(2):889–890. 10.1080/23802359.2017.1403865 PMC7799623 33474025

[CIT0016] Yu W, Yang Y, Qi Z, Shan B, Huang X, Liu Y, Lin H, Li T, Huang Z, Ma Z, et al. 2021. The complete mitochondrial genome of *Heniochus chrysostomus* (Perciformes, Chaetodontidae). Mitochondrial DNA Part B Resour. 6(3):933–935. 10.1080/23802359.2021.1888335 PMC7971298 33796688

[CIT0017] Yukai Y, Xiaolin H, Heizhao L, Tao L, Wei Y, Zhong H. 2019. The complete mitochondrial genome of *Chaetodon wiebeli* (Chaetodontiformes, Chaetodontidae). Mitochondrial DNA Part B Resour. 4(2):3145–3146. 10.1080/23802359.2019.1667894 PMC7706881 33365891

[CIT0018] Zhu K, Gong L, Lü Z, Liu L, Jiang L, Liu B. 2018. The complete mitochondrial genome of *Chaetodon octofasciatus* (Perciformes: Chaetodontidae) and phylogenetic studies of Percoidea. Mitochondrial DNA Part B Resour. 3(2):531–532. 10.1080/23802359.2018.1467218 PMC7799963 33474230

