# Supplementary material for: Analysis of the complete mitochondrial genome sequence of Japanese butterflyfish, Chaetodon nippon (Chaetodontiformes, Chaetodontidae)
Source: Mitochondrial DNA B Resour. 2023 Mar 13;8(3):389–92. doi: 10.1080/23802359.2023.2185080 (PMC10013452; doi:10.1080/23802359.2023.2185080)
Supplement: Supplemental Material [file TMDN_A_2185080_SM1263.docx]

**Analysis of the complete** **mitochondrial genome sequence of Japanese butterflyfish, *Chaetodon nippon* (Chaetodontiformes, Chaetodontidae)**

**Supporting data:**

**Figure S1.** Coverage with sequencing depth of *Chaetodon nippon* mitogenome.

**
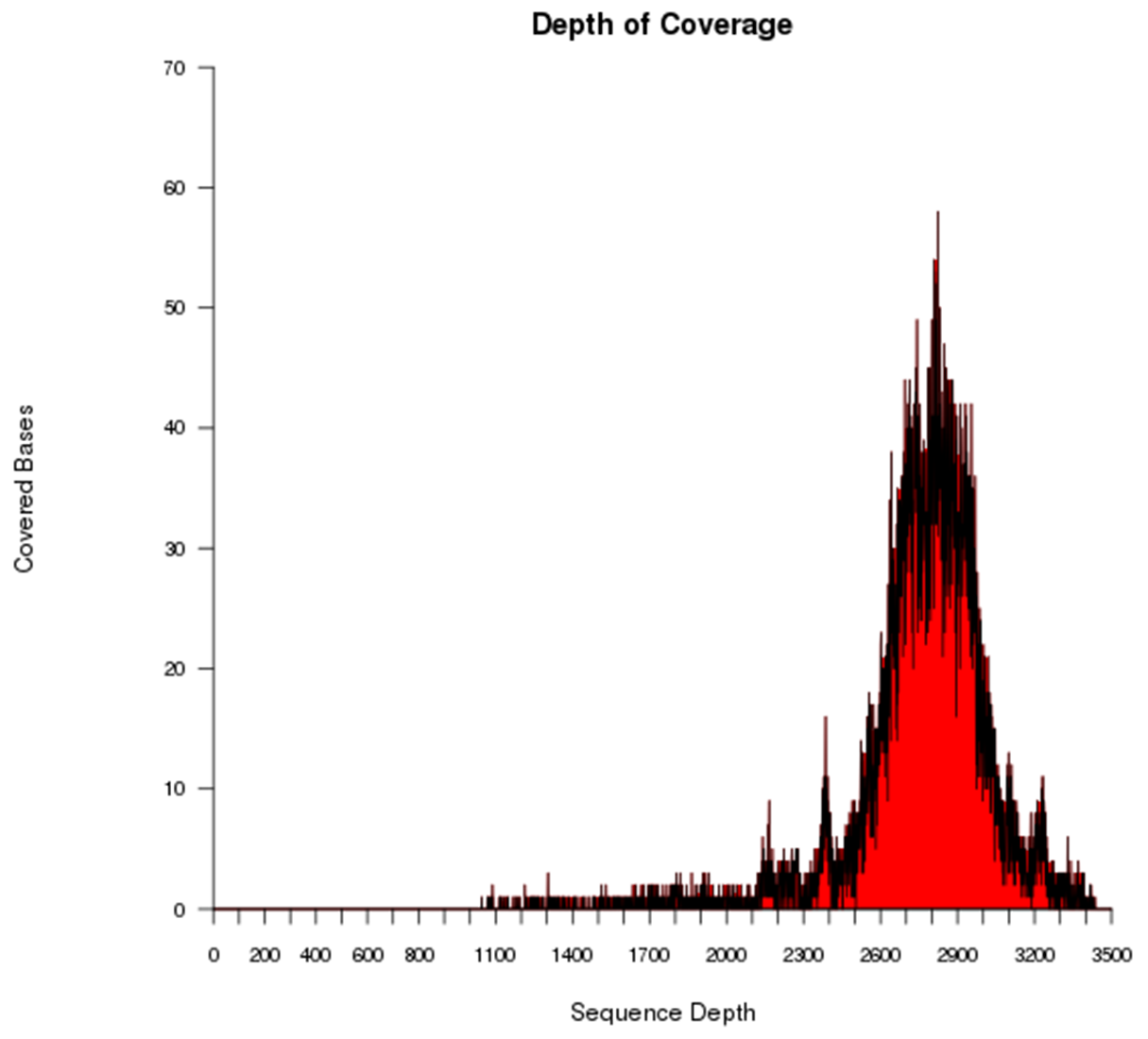
**

**Figure S2.** The phylogenetic tree using MrBayes based on the whole mitochondrial genome sequences of 12 species of Chaetodontidae and 03 species as outgroup members (*Leiognathus brevirostris* and *L*. *ruconius* from Leiognathidae and *Salvelinus malma* from Salmonidae). Numbers on branches are support values (maximum likelihood). The GenBank accession number is followed by the Genus and species name.


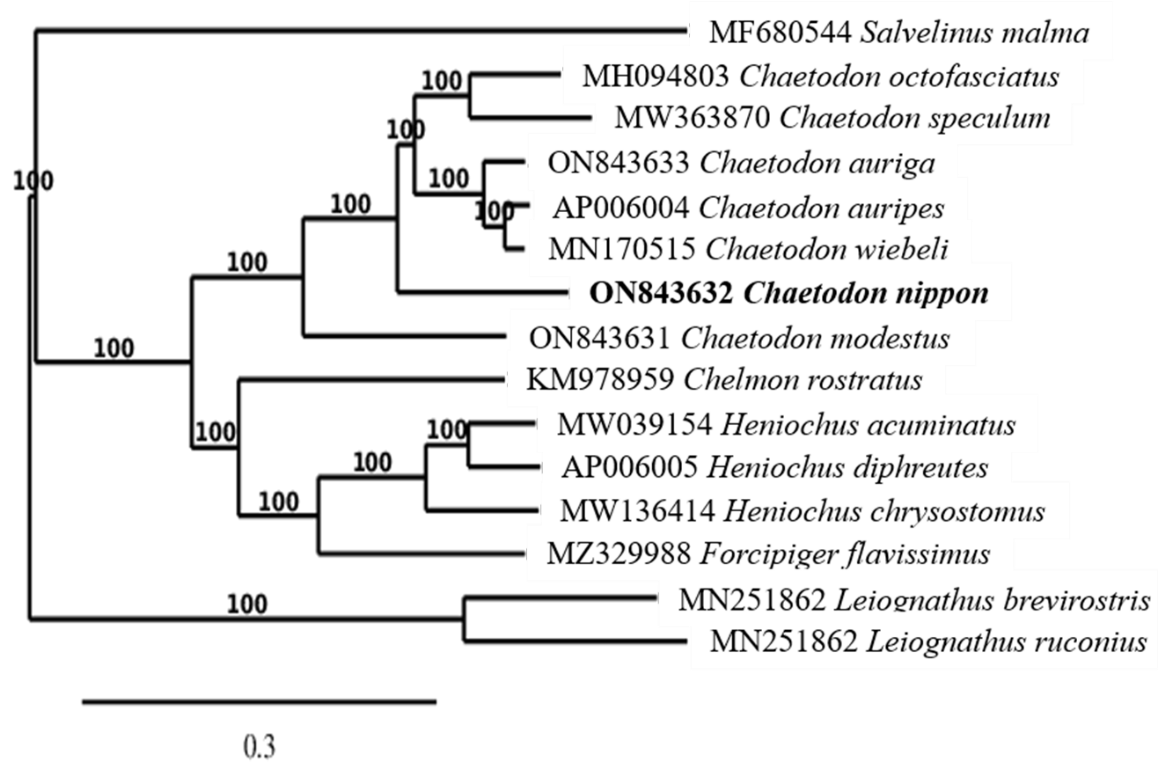


**Table S1.** Comparison of Mitochondrial Genome of several *Chaetodon* species.

| **Aspects compare** | ***C. wiebeli* (MN170515)** | ***C. auripes* (AP006004)** | ***C. auriga* (ON843633)** | ***C. speculum* (MW363870)** | ***C. octofasciatus* (MH094803)** | ***C. nippon* (ON843632)** | ***C. modestus* (ON843631)** |
| --- | --- | --- | --- | --- | --- | --- | --- |
| **Mitogenome Length** | 16,523 bp | 16,530 bp | 16,527 bp | 16,513 bp | 16,485 bp | 16,507 bp | 16,490 bp |
| **Base composition** | A: 4611 (27.91%),  T: 4366 (26.42%),  G: 2739 (16.58%),  C: 4807 (29.09%), and with a slight bias AT (54.33%) | A: 4607 (27.87%),  T: 4363 (26.39%),  G: 2738 (16.56%),  C: 4822 (29.17%), and with a slight bias AT (54.26%) | A: 4659 (28.19%),  T: 4353 (26.34%),  G: 2689 (16.27%),  C: 4826 (29.20%), and with a slight bias AT (54.53%) | A: 4595 (27.83%),  T: 4393 (26.60%),  G: 2715 (16.44%),  C: 4810 (29.13%), and with a slight bias AT (54.43%) | A: 4663 (28.29%),  T: 4520 (27.42%),  G: 2645 (16.04%),  C: 4657 (28.25%), and with a slight bias AT (55.71%) | A: 45645 (27.53%),  T: 4604 (27.90%),  G: 2767 (16.76%),  C: 4591 (27.81%), and with a slight bias AT (55.43%) | A: 4620 (28.02%),  T: 4732 (28.70%),  G: 2760 (16.74%),  C: 4378 (26.54%), and with a slight bias AT (56.72%) |
| **Total genes** | 22 tRNA, 13 PCG, 2 rRNA, and control region | 22 tRNA, 13 PCG, 2 rRNA, and control region | 22 tRNA, 13 PCG, 2 rRNA, and control region | 22 tRNA, 13 PCG, 2 rRNA, and control region | 22 tRNA, 13 PCG, 2 rRNA, and control region | 22 tRNA, 13 PCG, 2 rRNA, and control region | 22 tRNA, 13 PCG, 2 rRNA, and control region |
| **Total tRNA and PCGs on L strand** | 8 tRNA (Gln, Ala, Asn, Cys, Tyr, Ser, Glu, Pro) and 1 PCG (ND6) | 8 tRNA (Gln, Ala, Asn, Cys, Tyr, Ser, Glu, Pro) and 1 PCG (ND6) | 8 tRNA (Gln, Ala, Asn, Cys, Tyr, Ser, Glu, Pro) and 1 PCG (ND6) | 8 tRNA (Gln, Ala, Asn, Cys, Tyr, Ser, Glu, Pro) and 1 PCG (ND6) | 8 tRNA (Gln, Ala, Asn, Cys, Tyr, Ser, Glu, Pro) and 1 PCG (ND6) | 8 tRNA (Gln, Ala, Asn, Cys, Tyr, Ser, Glu, Pro) and 1 PCG (ND6) | 8 tRNA (Gln, Ala, Asn, Cys, Tyr, Ser, Glu, Pro) and 1 PCG (ND6) |
| **PCGs with incomplete stop codons** | ND2, COX2, COX3, ND3, ND4, CYTB | ND2, ATP6, COX3, ND3, ND4, CYTB | ND2, COX2, ATP6, COX3, ND3, ND4, CYTB | ND2, COX2, COX3, ND3, ND4, CYTB | ND2, COX2, COX3, ND3, ND4, CYTB | ND2, COX2, ATP6, COX3, ND4, CYTB | ND2, COX2, ATP6, COX3, ND3, ND4, CYTB |
| **Control region length** | 861 bp | 860 bp | 865 bp | 847 bp | 827 bp | 851 bp | 822 bp |
